# More than meets the eye: Understanding the importance of the materialities of care at the vaccination encounter in Portugal

**DOI:** 10.1177/13634593241313432

**Published:** 2025-01-18

**Authors:** Fábio Rafael Augusto, Ana Patrícia Hilário, Joana Mendonça

**Affiliations:** Instituto de Ciências Sociais, Universidade de Lisboa, Lisboa, Portugal; Centro Interdisciplinar de Ciências Sociais, Universidade de Évora (CICS.NOVA.UÉvora), Évora, Portugal; Instituto Universitário de Lisboa (ISCTE-IUL), CIS-Iscte, Lisboa, Portugal

**Keywords:** children, healthcare professionals, materialities, parents, vaccination

## Abstract

Caring practices during vaccination encounters are deeply interwoven with materiality, encompassing everyday objects and elements that play a crucial role for all actors involved. However, the significance of these materialities in shaping caring relationships within vaccination practices has been largely overlooked. This research seeks to fill that gap by exploring how mundane elements, such as the objects present during vaccination, contribute to the relational dynamics of the experience. Through a qualitative approach involving participant observation of vaccination encounters and interviews with 30 healthcare professionals, four key themes emerged: (i) objects as comfort devices, (ii) caring as gifts, (iii) reinvented medical instruments, and (iv) creating a friendly environment. These findings suggest that material elements are not merely passive tools but active “co-participants” in the vaccination process, influencing the interactions and emotional exchanges that occur. By acknowledging the role of materiality, this research enhances our understanding of vaccination as a relational experience, highlighting the importance of considering these often-overlooked factors in both practice and policy. The study offers valuable insights into how healthcare professionals can utilize materialities to foster more empathetic and supportive vaccination environments.

## Introduction

Childhood vaccination extends beyond a health procedure; it frequently emerges as a crucial moment for all those engaged in the process (Mishra and Graham, 2012). Indeed, vaccination can be a significant event, evoking negative feelings and emotions in the child, which subsequently concerns parents and prompts healthcare professionals to adopt strategies aimed at alleviating pain, fear, and anxiety (Cooper et al., 2021; Reich, 2016). As such, healthcare professionals assume a central and decisive role in cultivating a positive vaccination environment. They possess the opportunity to implement various care practices designed to ensure and enhance the child’s well-being (Taddio et al., 2010).

Caring practices within vaccination encounters are deeply intertwined with the concept of materiality, encompassing everyday objects and elements that hold significance for all actors involved in the process. Materiality serves as vital “clues” supporting healthcare professionals’ assessment of patient well-being and needs (van Hout et al., 2015). In this article, the term “materialities” refers not only to tangible objects, such as medical tools and comforting items (Cleeve et al., 2020), but also to intangible attributes, such as the surrounding environment and overall atmosphere (Buse et al., 2018). This broad definition will help us to make previous assumptions about the materialities that could be of importance in the vaccination encounter (Buse et al., 2018). Previous research has underscored the social and symbolic significance of seemingly mundane practices for both patients and family members, contributing to the negotiation of important caring moments (Ellis, 2018). However, there remains a notable gap in understanding the role of materialities involved in vaccination practices and their impact on shaping caring relationships within this context.

This study seeks to address this gap by closely examining vaccination encounters, aiming to enhance comprehension of the care interactions during childhood vaccination. Portugal serves as an especially pertinent context for this analysis, given its high levels of vaccine confidence (Larson et al., 2018) and strong vaccination coverage^1^ (Direção-Geral de Saúde, 2020), alongside an increase in vaccine hesitancy concerning childhood immunization (George et al., 2017). Employing a qualitative methodology, this study focuses on analyzing the encounters facilitated by childhood vaccination, exploring how they are shaped by mundane materialities and the emergence of care relationships. Care itself manifests in diverse forms, encompassing not only physical work or a form of labor (“caring for”) but also emotional investment, empathy, and concern (“caring about”) towards others (Abbots et al., 2015; Rummery and Fine, 2012). By centering on mundane materialities and examining their (re)appropriation by social actors, it enables us to shed light on the intricacies of care provision (Buse et al., 2018).

Unravelling the complexities of the “materialities of care” requires a holistic understanding of the interconnections between materials, practices, and socio-cultural contexts (Chapman, 2006). The term “materialities,” encompasses tangible elements, and integrates “spatial elements, objects, and bodies” (Cleeve et al., 2020: 127). Beyond merely physical objects, “materialities” extend to the interface between the material and the immaterial, where intangible qualities such as environment and atmosphere come to the fore (Buse et al., 2018). The intricate interplay between materialities and care practices underscores the nuanced nature of caregiving, where tangible and intangible elements intertwine to shape the caregiving experience (Cattell et al., 2008; Martin et al., 2015; Milligan and Wiles, 2010; Williams, 1998). By directing attention to various mundane elements, such as the objects present during vaccination, this research contributes to an enhanced understanding of vaccination as a relational experience. Additionally, it suggests new avenues for future sociological research on vaccination practices, offering insights into the interplay between materialities, care practices, and patient experiences within healthcare settings.

## Background

Childhood vaccination usually implies clinical encounters between the child, their parents and the healthcare professional who performs the inoculation. Within these encounters, the child’s healthy and “pure body” is given a shot of an artificial agent that prevents the risk of disease (Mishra and Graham, 2012; Reich, 2016). The lurking needle can lead to feelings of pain and anxiety in the child and in their parents, who fear for their child’s physical integrity (Mishra and Graham, 2012). Healthcare professionals hold a central role in these encounters, wielding the “clinical authority” to guide the immunization process. Their conduct during these interactions is pivotal, capable of either exacerbating or alleviating vaccine-related anxiety. Notably, healthcare professionals’ engagement in strategies to minimize pain and anxiety during childhood vaccination has been shown to effectively prevent healthcare avoidance behaviors stemming from needle fear. These behaviors, such as non-adherence with vaccination schedules, underscore the importance of healthcare professionals’ involvement in ensuring a positive vaccination experience and fostering trust among parents in healthcare providers (Taddio et al., 2010).

Although sociological research on this subject is still limited (Lindén and Odenbring, 2022), there is evidence about the influence of caring practices on vaccination attitudes. These caring practices may serve as a protective shield against fear and anxiety-inducing experiences, such as the needle prick (Lindén and Odenbring, 2022; Mishra and Graham, 2012). However, the extent to which healthcare professionals can incorporate these practices is contingent upon various factors, including their prior training in pain management strategies (Taddio et al., 2010) and the demands placed on them by their workload (Rockliffe et al., 2020). Lindén and Odenbring (2022) proposed the concept of “modes of doing good” to analyze mundane practices of “good care” in the specific case of childhood vaccination against human papillomavirus (HPV) carried out at schools. These practices included performing the vaccination without haste, contributing to a relaxing and calm environment, informing the children and replying to their concerns previous to the vaccination and allowing them to bring a friend for support. These routines are often “invisible,” as they are mainly focused on knowledge and informed consent as strategies to mitigate children’s anxiety regarding the needle pricking. The modes of “doing good care” have also been identified in similar studies, which have explored for instance prenatal testing (Schwennesen and Koch, 2012).

The perception of vaccination as merely a straightforward medical intervention aimed at disease prevention has significantly contributed to the neglect of its material dimensions. This narrow view overlooks the complex interplay of objects, environments, and interactions that constitute the practice of vaccination. By focusing solely on the biomedical aspects—such as vaccine efficacy and immunological response—there is a tendency to ignore the broader material context in which vaccinations occur (Kamp et al., 2023; Rodrigues and Plotkin, 2020). Despite their seemingly mundane nature, these materialities play a pivotal role in shaping the dynamics of caring relationships within vaccination contexts. Aligned with Lindén and Odenbring’s (2022) concept of “modes of doing good,” analyzing these materialities offers insights into enhancing childhood vaccination experiences, potentially transforming them into more positive encounters. Consequently, viewing vaccination as both a material and relational experience opens up new analytical possibilities (Montgomery et al., 2024). Mundane material elements harbor significant relational and affective potential, actively contributing to the vaccination process (Bennett, 2007). This potential can only be fully realized under the premise that material goods, with their fluid and interactive nature (Buse et al., 2018), constitute essential elements of caregiver-patient relationships (Ellis, 2018; van Hout et al., 2015), thereby influencing the overall vaccination experience. Indeed, materialities encompass both visible and invisible elements, from tangible objects to intangible aspects like emotions and interpersonal dynamics (Ellis, 2018; Martin et al., 2015). Within this framework, mundane materialities transcend their functional roles to become conduits for empathy, trust, and emotional connection (Cleeve et al., 2020; Ivanova et al., 2016).

Understanding vaccination as both a material and relational experience broadens the discourse beyond clinical procedures, prompting exploration into the complex tapestry of human connection and compassion (Pala, 2023). In essence, the materialities of vaccination could not only facilitate the technical aspects of the process but also serve as catalysts for deeper, more meaningful engagements. They embody the silent yet tangible essence of caregiving, enriching the vaccination journey with empathy and understanding. Moreover, it is crucial to recognize that the care practices within the context of vaccination are unique and dynamic, with profound implications for all entities involved, both human and non-human. The act of vaccination weaves together the physical and emotional realms, highlighting the interconnectedness of our world (Puig de la Bellacasa, 2011).

Drawing on Latimer (2018) work, we argue that paying attention to the mundane things in vaccine care encounters would help to expand our understanding of unnoticed aspects that may influence the outcomes of the vaccination encounter. Previous research has convincingly shown that materialities are part of how relationships are framed in health and care practices (Brownlie and Spandler, 2018; Buse and Twigg, 2018). For instance, mundane objects such as gloves have been outlined to provide tacit, embodied and sensory ways of knowing to healthcare professionals (Pink et al., 2014). The current study aims to respond to the call of Maller (2015) for a social practice-based approach that would enable us to reflect on how materiality could offer valuable insights to explore the moments of “doing” in healthcare practices (Buse et al., 2018), using the vaccination encounter as an example.

## Methods

The research presented here represents the culmination of a qualitative study conducted in Portugal, unfolding through two meticulously planned phases. Initially, spanning from November 2021 to March 2022, the research team immersed themselves in participant observation across various healthcare settings, including a healthcare center, a private practice and a hospital located in the metropolitan area of Lisbon. This observation keenly aimed to unravel the intricate dynamics of interaction among healthcare professionals, parents, and children (ranging from 2 months to 6 years old) during the vaccination process. Transitioning into the second phase, the research team conducted interviews with 30 healthcare professionals. These interviews were crafted to delve into the nuanced aspects of the interaction at the vaccination encounter from the perspective of healthcare professionals, particularly focusing on the strategies employed to address vaccine hesitancy^2^. Conducted in Portuguese and recorded with participants’ permission, the interviews were subsequently verbatim transcribed. Ethical clearance for this study was obtained from the hosting institution’s Ethics Committee, as well as the places where fieldwork took place. Informed written consent was obtained from all participants, on the understanding that their participation was voluntary and that all collected data underwent meticulous anonymization procedures. Regarding data analysis, all research materials, namely interview transcripts and field notes were anonymized and then imported into Nvivo. Employing a thematic analysis approach as suggested by Braun and Clarke (2006), the research team embarked on a systematic process. Beginning with an open-coding procedure, first-order themes were iteratively refined into more nuanced categories. Through comprehensive scrutiny, these refined themes were distilled into core themes. Consequently, four distinct themes emerged: (i) objects as comfort devices; (ii) caring as gifts; (iii) reinvented medical instruments; (iv) creating a friendly environment. The themes will be presented in the following section and selected quotations either from fieldwork or interviews will be used in a complementary way.

## Findings

As mentioned previously, this section aims to present the primary findings from our fieldwork. Aligned with the themes identified through the thematic analysis, our results illuminate various aspects. Firstly, we discuss how the presence of certain objects during the vaccination encounter can significantly enhance the comfort of all participants, particularly focusing on the child’s experience. Following this, we delve into the exploration of gift donation practices as a strategic approach to mitigate the inherent tension often associated with vaccination procedures. Additionally, our findings shed light on innovative adaptations of medical instruments tailored to meet the unique needs of children. Lastly, we emphasize the central role of the vaccination environment, highlighting elements such as color and background music, which can profoundly influence the overall experience for everyone involved.

### Objects as comfort devices

Throughout the fieldwork, numerous strategies were identified, employed by both parents and healthcare professionals, to enhance the child’s vaccination experience. Among the primary artifacts utilized by these individuals are toys, including stuffed animals, Legos, and action figures. These items are selected for their familiarity and ability to provide comfort to children, making them integral components of healthcare settings. By incorporating these comforting objects, healthcare providers and parents can significantly reduce children’s anxiety and create a more positive and supportive environment during vaccinations.

The nurse assured that the vaccination “won’t hurt at all” and promised to apply a “magic” spray with a banana scent. The mother commented, “only big girls get vaccines.” The girl brought a stuffed panda to the appointment, and her mother explained that it was “for comfort.” (Field Note, Healthcare Center)

The baby received the vaccination while lying on the stretcher, with his father standing by, holding him, gripping his hand, and distracting him with a toy. (Field Note, Hospital)

These excerpts vividly illustrate the proactive role parents play in using toys to distract their children from the discomfort and anxiety often associated with vaccinations. Moreover, healthcare professionals have also adopted this effective strategy, demonstrating their adaptability and sensitivity to the child’s needs during the vaccination process. This collaborative approach between parents and healthcare providers underscores a shared commitment to creating a less stressful vaccination experience for children.

The nurse entered the scheduled vaccines into the computer and, using the panda as a visual aid, explained the upcoming procedure for the child’s arm. (Field Note, Healthcare Center)

Another prevalent strategy observed was the utilization of videos as a distraction technique. Both parents and healthcare professionals used digital devices, such as computers, tablets, or mobile phones, to engage and entertain the child during the vaccination procedure. Typically, these chosen videos feature beloved and familiar cartoon characters, serving as a comforting and captivating distraction for the child amidst the vaccination process. This approach not only provides immediate relief from anxiety but also fosters a positive association with the vaccination experience. By incorporating these digital distractions, caregivers and healthcare professionals can create a safer, more calming environment for children during vaccinations.

The 10-year-olds no longer find painting as amusing; it’s no longer their preferred activity. Instead, I’m beginning to introduce alternative options. I might say, “Let’s wrap this up quickly, and then you can watch a game on YouTube.” These adjustments cater to their evolving interests and preferences. (Palmira, Nurse)

The mother began humming a song that the babies enjoyed, prompting the nurse to immediately play that song on YouTube. Consequently, the vaccination process consistently occurred with children’s music in the background. (Field Note, Hospital)

The nurse inquired about the girl’s preferences for videos or music, to which she responded that she enjoyed watching “Elsa” [a fictional Disney character]. Subsequently, the nurse played an “Elsa” song on YouTube to provide entertainment for the girl during the vaccination. (Field Note, Healthcare Center)

These strategies have proven to be exceptionally effective for several reasons. Firstly, they leverage familiar devices that children interact with daily. Whether it’s smartphones, tablets, or computers, these gadgets are integral parts of children’s lives, fostering a sense of comfort and ease during stressful situations like the vaccination encounter. Moreover, children possess the requisite skills to navigate and engage with these technological artifacts across various contexts, empowering them to seamlessly interact with them during the vaccination procedure. This familiarity and proficiency significantly facilitate the use of these devices, ensuring smooth and successful implementation without encountering any significant hurdles.

Despite the potential benefits, reliance on digital mechanisms doesn’t always yield the desired results. In certain situations, healthcare professionals may find themselves deeply affected by a child’s distress, making it difficult to effectively manage the situation. The emotional impact of witnessing a child’s suffering can be profound, sometimes leaving healthcare providers feeling overwhelmed or inadequate in their ability to alleviate the child’s discomfort. This emotional strain underscores the complexity of paediatric care, highlighting the need for comprehensive strategies that address both the physical and emotional well-being of young patients.

It’s challenging for me. . . really challenging to administer all those vaccinations to a one-year-old child. I’ll be honest about it. I handle them gently, but still, I believe the child senses. . . [. . .] I try everything—put on their favorite cartoons, bring out the toys—but even then, it’s heartbreaking. (Susete, Nurse)

This emotional burden can impede their capacity to respond effectively, despite the availability of digital tools. It underscores the nuanced challenges inherent in paediatric care, where empathy and emotional resilience are crucial components alongside technical skills. As such, fostering a supportive environment that acknowledges and addresses the emotional well-being of healthcare professionals is essential for optimizing patient care outcomes. By supporting the mental and emotional health of those administering care, we can ensure a more compassionate and effective approach to paediatric healthcare.

### Caring as gifts

Care practices related to gift-giving emerged as noteworthy observations. Beyond the clinical procedures, healthcare providers engaged in the thoughtful gesture of offering various items to children. These included not only toys but also books, informative leaflets, and printed drawings, tailored to the child’s age and interests. This personalized approach aimed to not only alleviate immediate distress but also to foster a positive and supportive environment during the vaccination process. By incorporating elements of play and education, these gestures extended beyond mere medical interventions, highlighting the holistic approach taken towards paediatric care.

The mother reported that her youngest son was experiencing constipation, prompting the doctor to request that she undress the child for examination. During this time, the doctor went to retrieve a book from the closet to offer to the older child, who remained upset. Once found, the doctor handed the book to the child, and a woman accompanying them began reading, successfully soothing the distressed child. (Field Note, Private Practice)

These expressions of affection are typically highly valued by both parents and children. The prospect of a reward for “good behavior” related to the vaccine administration motivated numerous children to overcome the fear and apprehension expressed during the initial consultation phase. This incentive-based approach not only encourages cooperation but also reinforces positive behavior, making the vaccination experience more manageable and less intimidating for young patients.

After completing the procedure, the mother promptly dresses the baby, while the head nurse fetches a toy to distract the crying infant. (Field Note, Hospital)

The use of drawings is particularly fascinating due to the high level of engagement it fosters and its unique ability to facilitate communication with children in a creatively distinct manner. Unlike verbal or written communication, drawings provide a visual medium through which children can express their thoughts, feelings, and concerns more freely. This form of visual language transcends linguistic barriers, making it especially effective in situations where verbal communication may be limited, such as with younger children or those with communication difficulties. By encouraging children to draw, parents and healthcare professionals can gain deeper insights into the child’s emotional state and concerns, fostering a more comprehensive and empathetic approach to paediatric care.

What I do now for five-year-old children is always keep a collection of drawings and paintings on my computer. I have them sorted here: cars, bunnies, dinosaurs, Frozen characters, and more. I’m continuously updating the collection; for instance, I plan to add animals soon, as some were unfamiliar to me. It’s interesting how children’s preferences evolve; I hadn’t heard of PJ Masks until they became popular with my own kids. Then there were LOL dolls—I had to look those up! So, I try to stay current with what they enjoy. Here’s my process: a few days before their appointment, I select a drawing and print it out. I prepare pencils and pens, although we’ve had to limit shared items due to Covid. Still, we try to give them some playtime. I show the drawing, and often they’re delighted. I’ll encourage them, saying something like, “Be as strong as the princess or the superhero.” At the age of 10, they’re a bit more sceptical, but at 5, you can still inspire them (chuckles). (Palmira, Nurse)

The nurse engaged with the child in a warm and friendly manner, offering a blank sheet of paper and a pen for drawing while posing questions to the parents. [. . .] The nurse inquired if the child was interested in drawing the vaccine, highlighting its role as a “shield against all diseases,” and also asked about the child’s favourite cartoon. (Field Note, Healthcare Center)

In the last excerpt, it is evident that drawings serve to communicate positive messages about vaccination. The nurse skillfully utilizes the imagery of the impending vaccine as a shield, effectively highlighting its central purpose in shielding the child from potential diseases. This analogy not only simplifies the concept of vaccination but also imbues it with a sense of purpose and protection, instilling confidence in both the child and their caregivers. Furthermore, by likening the vaccine to a shield, the nurse not only emphasizes its defensive function but also imbues it with a sense of strength and resilience. This strategy serves to empower the child, portraying them as active participants in the management of their own health and well-being. It reframes the vaccination experience from one of fear or uncertainty to an act of bravery and fortitude, fostering a positive and proactive mindset towards immunization. Moreover, the use of such visual metaphors facilitates comprehension and retention, particularly among young children who may struggle with abstract concepts. By presenting vaccination as a tangible and relatable concept through drawings, healthcare providers can demystify the process and allay anxieties, making it more accessible and comprehensible for children. Overall, the strategic use of drawings to convey positive messages about vaccination not only educates and reassures but also empowers children to take an active role in protecting their health. It underscores the importance of creative and empathetic communication strategies in paediatric healthcare, ultimately contributing to a more informed and cooperative approach to immunization.

### Reinvented medical instruments

The study unveiled a fascinating trend: certain medical instruments underwent creative transformations to better accommodate the needs and sensibilities of children. Notable examples included the ingenious conversion of a medical stretcher into a friendly dinosaur and the use of cartoon bandages. These adaptations went beyond mere functional modifications; they were deliberate efforts to make medical experiences more approachable, engaging, and comforting for young patients. Take, for instance, the transformation of the medical stretcher into a playful dinosaur. This imaginative redesign not only served its practical purpose of transporting children but also ignited a sense of wonder and delight. By incorporating elements from children’s imagination, such as dinosaurs, healthcare providers effectively transformed an otherwise daunting medical device into a reassuring and whimsical companion. This not only helped alleviate fear and anxiety but also sparked children’s creativity, turning what could have been a frightening experience into an exciting adventure. Similarly, the use of cartoon bandages showcased how everyday medical supplies were creatively repurposed to create a more positive and reassuring environment for children. Instead of bland and clinical bandages, colorful designs featuring beloved cartoon characters adorned wounds. These bandages not only provided effective wound care but also served as a source of comfort and distraction, turning a routine aspect of medical care into a fun and enjoyable experience for children.

The mother mentioned bringing adhesive bandages with cartoon characters because she was unsure if the healthcare center would provide ones with designs or only “plain” ones. (Field Note, Healthcare Center)

By assigning alternative meanings to specific medical elements, children were able to perceive them as less intimidating and frightening. This approach proved invaluable in the context of vaccination, where children often experience heightened anxiety and negative emotions. By transforming medical instruments into familiar and friendly objects, healthcare providers helped children feel more at ease during these tense moments, fostering a sense of security and empowerment. Furthermore, this process of appropriating healthcare instruments can manifest itself at various levels, ranging from simple modifications to elaborate transformations. Whether it’s a creative redesign of a medical device or the incorporation of playful accessories, the objective remains consistent: to create a more child-friendly and welcoming healthcare environment. Ultimately, these adaptations not only enhance the overall quality of care but also cultivate trust, comfort, and resilience among young patients and their families.

When the nurse left the office to retrieve the vaccines, the girl’s mother attempted to engage her by exploring the child development scale displayed on the wall. She conducted exercises with her daughters that aligned with the skills described for their respective ages. (Field Note, Healthcare Center)

In the described scenario, the mother ingeniously utilizes a healthcare artifact to redirect her daughters’ attention away from the vaccination process. While the object itself remains unchanged in its physical form and institutional definition, its purpose undergoes a subtle yet transformative shift. Rather than serving its conventional function within the healthcare setting, it becomes a strategic tool employed by the mother to mitigate the potentially negative impact of the vaccination experience on her daughters. This demonstrates a remarkable example of resourcefulness and adaptability in navigating challenging situations. By leveraging the artifact as a pretext, the mother effectively reframes the vaccination process for her daughters, transforming what could be perceived as a daunting or distressing event into a more manageable and even enjoyable moment. In doing so, she harnesses the power of distraction and diversion to ease any apprehension or anxiety her daughters may be experiencing, thereby fostering a more positive and supportive environment for the vaccination procedure. Moreover, this approach highlights the importance of creativity and improvisation in caregiving. While the artifact itself may not undergo physical alterations or redefinition, its role is dynamically reimagined to meet the specific needs and circumstances of the moment. This underscores the flexibility and adaptability required of caregivers in responding to the unique needs and preferences of their children, particularly in healthcare settings where emotions may run high. Overall, the scenario exemplifies the creative and resourceful ways in which caregivers can utilize healthcare artifacts to navigate challenging situations and support their children’s well-being. By recognizing and harnessing the potential of everyday objects in novel ways, caregivers can play a crucial role in alleviating stress and promoting comfort and resilience in their children, even during potentially difficult moments like vaccinations.

### Creating a friendly environment

The setting where care practices for vaccination take place is profoundly influenced by sounds and ambient music. These auditory elements play a crucial role in shaping the dynamics of relationships and, most importantly, in molding the child’s perception of the various processes and instruments associated with vaccination.

Throughout the administration of the three vaccines, the parents sang familiar children’s songs, a customary practice in soothing the baby during such procedures. (Field Note, Hospital)

The father then turned on his cell phone, playing a children’s song video, and placed it in front of the boy while the doctor continued with the examination. (Field Note, Hospital)

The nurse announced the administration of the second vaccine while playing soothing baby music on the computer. She suggested that the mother could continue playing this music at home, though it wouldn’t replace the comforting effect of her voice. Despite the baby crying intensely during the vaccine administration, the nurse and the doctor maintained their communication in a gentle and soft tone. (Field Note, Healthcare Center)

While the sounds generated, either by objects or human interaction, didn’t consistently yield positive effects on the child, there were instances where they actively contributed to fostering a calming environment. As evidenced in the provided excerpts, a synergy exists among various care practices that leverage sound to aid the child in navigating the vaccination experience. These include the utilization of gentle music via electronic devices and the employment of a soothing tone by both parents and healthcare professionals.

The data collected not only underscores the significance of auditory elements in vaccination settings but also sheds light on the importance of other environmental factors that contribute to the overall experience. Beyond sounds and ambient music, elements such as decorative colors, games, and quizzes emerge as crucial components in crafting a friendly and supportive environment for vaccination processes.

We transformed our vaccination room into a vibrant, child-friendly space on-site. Our goal was not just administering vaccinations but creating an environment conducive to children’s comfort and distraction. The walls were adorned with colourful murals, engaging games, and interactive crosswords. Ultimately, we aimed to alleviate any anxieties and make the vaccination process a more positive experience for our young patients. (Pilar, Nurse)

These elements serve as powerful tools in mitigating anxiety, fostering a sense of familiarity, and redirecting attention away from the discomfort associated with vaccinations. Decorative colors, carefully chosen to evoke feelings of warmth and reassurance, transform clinical spaces into inviting sanctuaries. They not only stimulate positive emotions but also help alleviate the sterile ambiance often associated with medical settings. By creating visually appealing surroundings, healthcare providers can establish an environment conducive to relaxation and trust, essential for successful vaccination outcomes. Integrating interactive games and quizzes into the vaccination process offers more than mere distraction; it promotes engagement and empowerment. Through playful activities tailored to children’s interests and developmental stages, healthcare professionals can empower young patients to actively participate in their healthcare journey. These interactive elements not only alleviate fear but also foster a sense of agency, enabling children to feel more in control of their vaccination experience.

Additional strategies aimed at cultivating a positive vaccination environment revolved around the power of tactile experiences. Indeed, numerous accounts highlighted the significance of physical contact, particularly being held on a parent’s lap during vaccination, as a potent means of alleviating fear and anxiety. Moreover, various forms of touch were identified as effective tension-relieving techniques, with gentle tickling being notably mentioned.

To help soothe their fears, I find it effective to gently seat them, perhaps on their mother’s or father’s lap, or even on a nearby chair, tailored to their activity. From there, I initiate a playful tickling session, which often works wonders in easing their anxiety. (Palmira, Nurse).

By integrating these tactile strategies into vaccination practices, healthcare providers can tap into the innate human need for physical connection and comfort, ultimately fostering a more positive and supportive environment for both children and their caregivers.

Promoting a positive vaccination environment presents challenges that aren’t always surmountable. Extensive fieldwork has illuminated numerous instances where the physical setting designated for vaccinations failed to meet the criteria for an optimal environment. These shortcomings, ranging from inadequate facilities to logistical constraints, have consistently impeded the efforts of healthcare professionals striving to foster a warm and accommodating atmosphere conducive to vaccination acceptance and engagement.

It would greatly benefit our facility to incorporate a designated waiting area tailored for children. This space would not only alleviate the boredom of waiting, even if the wait is brief, but also create a more pleasant experience overall. Imagine if we could implement such improvements under different circumstances. Unfortunately, the constraints imposed by COVID have hindered our progress. Nonetheless, envision a waiting room equipped with engaging activities such as musical instruments – perhaps a xylophone – to captivate children while they await their vaccinations. Such enhancements would undoubtedly enhance the overall experience for both children and caregivers. (Paulina, Nurse).

Our current facilities, being containers, serve as temporary spaces where sound easily travels from one room to another. Unfortunately, this setup occasionally leads to unsettling experiences, as the sound of medical procedures can carry across the rooms, creating distressing impressions. Clearly, this situation is far from ideal. (Sara, Nurse).

One of the prominent challenges faced involves the inadequacies in the physical infrastructure of vaccination sites. Issues such as the absence of a designated waiting room and concerns arising from insufficient isolation facilities gradually degrade the environment and operational efficiency of the space. The absence of a dedicated waiting area can lead to congestion and discomfort, while inadequate isolation facilities may compromise privacy and increase the risk of cross-contamination. These infrastructural shortcomings not only affect the efficiency of the vaccination process but also have a substantial impact on the experiences of healthcare professionals, parents, and children.

## Discussion

The materialities of routine practices in vaccination often go unnoticed due to their ordinariness (Kamp et al., 2023). We took as a point of departure that these materialities are key in the construction of caring relationships (Ellis, 2018; van Hout et al., 2015) within the vaccination context. Aligned with the notion of “modes of doing good” (Lindén and Odenbring, 2022), we ground our study on the belief that mundane materialities are important instruments for analyzing care practices within the vaccination encounter. The focus on mundane materialities may help to highlight some practices that may be of value to manage childhood vaccination. Vaccination has been theorized in ways that mundane materialities have not been made visible (Pala, 2023) and thereby the current study intended to address this gap.

The findings revealed that mundane materialities significantly influence the interactions occurring during childhood vaccination. By examining the everyday objects present in the vaccination encounter, this article enhances our understanding of vaccination as a relational experience (Montgomery et al., 2024). It underscores how both tangible items and intangible elements, such as the overall environment and atmosphere, play crucial roles in supporting children through the vaccination process, especially in alleviating the stress associated with needle pricking (Bell, 2018; Nettleton et al., 2020; Pink et al., 2014). The study highlights how seemingly ordinary objects, alongside the ambient conditions of the vaccination environment, contribute to a more supportive and less intimidating experience for children. This focus on materialities not only deepens our insight into the relational dynamics of vaccination but also opens new avenues for future sociological and anthropological research on vaccination practices. By drawing attention to these elements, the article suggests that future studies could further explore how material and environmental factors impact the effectiveness and emotional experience of vaccinations.

Having identified certain artifacts as particularly important during the vaccination encounter, this article showed how they act relationally in the context of childhood vaccination (Bennett, 2007). The relational effect that mundane materialities can have on the experience of vaccination is demonstrated in the findings presented alongside this article. These materialities are often used by both healthcare professionals and parents to divert the child from the anxiety and pain associated with vaccination. Furthermore, inviting children to create drawings about their vaccination experience—depicting the objects, individuals, and emotions involved—not only promotes their agency in the process but also carries significant symbolic value. The use of familiar objects such as toys, videos, songs and cartoon bandages give the child a sense of security, making the consultation environment more welcoming and less threatening. The inoculation simulation on a child’s favorite stuffed panda allows the child to foresee what will happen, making him feel more in control of the situation. In this example, although the nurse was not expecting the child would take his teddy to the vaccination appointment, she promptly used it as a relational object. This is in line with our finding that caring practices are used either spontaneously or in a planned manner (Buse et al., 2018). This is probably due to the unpredictability about the dynamics between the various actors in the vaccination encounter, which are influenced by factors such as the child’s personal characteristics, parents’ involvement in the vaccination process and the objects made available to healthcare professionals.

Therefore, we claim that these materialities should be understood as “co-participants” (Bennett, 2007) in the vaccination process. Nevertheless, because of their ordinariness these materialities are often unnoticed (Cleeve et al., 2020). The findings are aligned with previous work, which have demonstrated how material things are key in carer-patient relationships (Ellis, 2018; van Hout et al., 2015). The interactive and fluid nature of material “things” (Buse et al., 2018) are made apparent in the findings described in our study. The findings reveal that certain material practices such as bringing a toy to the vaccine encounter should not be acknowledged as mundane (Latimer, 2018). Indeed, these material practices are important occasions of care. The findings are very much aligned with Puig de la Bellacasa (2011) argument that “engaging with care requires” a “commitment to neglected things” (p. 85). This is in line with previous research by Cleeve et al. (2020), who have acknowledged the impact of the organization of professional care on the ways in which the materialities of care are understood. The “neglected things,” namely the basic caring tasks in the vaccination encounter, are important occasions for caring (Latimer, 2018). Materialities permeate practices of care, as well as care permeates materialities in a relational way (Buse et al., 2018). The findings suggest that materialities are constitutive of the relationships that take place at the time of vaccination (Brownlie and Spandler, 2018).

Whereas material culture has been deeply explored within the sociology of health and illness, namely the role of technological innovation, less attention has been paid to other aspects of material culture (Buse et al., 2018). Extending this work helps to understand how material practices can affect the vaccination encounter. We argue that it is key to acknowledge the importance of ordinary objects and elements that often go unnoticed (Kamp et al., 2023) in the vaccination encounter as this has an impact on how the vaccination process occurs and in the interaction between the different actors (i.e. children, parents and healthcare professionals). The findings convincingly show that materialities generate actions and reactions (Martin et al., 2015) that could have an impact on the outcomes of the vaccination encounter.

By placing particular emphasis on these moments of interaction between care and materialities, we aim to uncover the deeper layers of meaning and significance that emerge. It is within these intersections that we find rich insights into the complexities of care provision, shedding light on how material objects, spaces, and environments shape and are shaped by caregiving practices (Buse and Twigg, 2018; Puig de la Bellacasa, 2011). Moreover, we challenge conventional understandings of the vaccination encounter by turning our attention to the seemingly mundane and taken-for-granted aspects of caregiving (Abbots et al., 2015). By interrogating these overlooked dimensions, we seek to unearth new perspectives and fresh understandings of care practices. This approach invites us to reconsider and reevaluate established frameworks, opening up avenues for innovative thinking and transformative insights. The current article offers a broader understanding of the vaccination encounter, weaving together threads of practice, materiality, and meaning. The article aligns with the new materialist turn within the social sciences (Brown, 2004; Chapman, 2006; Fox, 2016), underlining the need to pay attention to the “neglected things” (Puig de la Bellacasa, 2011) as this would help to increase our understanding of what are “important for occasions to actually be caring” (Latimer, 2018: 379) at the vaccination encounter.

## Limitations of the study

This study presents two notable limitations that merit discussion. First, many of the findings are based on inferences made by the researchers and healthcare professionals interviewed, rather than direct input from children and their parents. While these conclusions are grounded in extensive observation and interaction within the study context, they rely on interpreted behaviors and staff feedback rather than firsthand accounts. For instance, it was inferred that elements like colorful walls or cuddly toys reduced anxiety, yet this assessment lacks validation from the perspectives of the children or their caregivers. This reliance on inferred interpretations introduces a degree of subjectivity, as the findings primarily reflect the professional judgments and observations of those present. Although these insights are valuable, a more comprehensive understanding could be achieved by incorporating direct testimonials from children and parents. Future research should address this gap by employing methodologies that amplify the voices of these key stakeholders, thereby enhancing the robustness and depth of the findings. Secondly, while the study aims to analyze the various materialities—both tangible and intangible—that help mitigate the pain and potential trauma of the vaccination process, the main findings often converge on a central theme: the use of distraction strategies in healthcare settings. This focus may create an impression of thematic overlap or redundancy, as the nuances of distraction techniques are explored across different contexts. Nevertheless, this approach was intentionally chosen to emphasize the multifaceted nature of the identified distraction strategies and their material dimensions. By examining these strategies through the lens of materialities, the study sheds light on the diverse ways in which objects, environments, and intangible attributes contribute to alleviating anxiety and discomfort. However, it is acknowledged that an alternative analytical focus—shifting from materialities to distraction strategies as the primary framework—could also be a valid approach. Such a perspective might allow for a more streamlined discussion while maintaining the depth and significance of the findings.

## Conclusions

Currently, our understanding of how mundane material elements shape childhood vaccination practices remains limited. This study endeavors to address this knowledge gap by meticulously examining the nature and execution of caring practices across diverse vaccination settings, with a particular focus on their implications for all involved stakeholders, especially the child.

Our findings highlight the critical role that mundane materialities play in shaping the vaccination experience. By analyzing these elements, we demonstrate how they impact interactions during childhood vaccinations. Healthcare professionals and parents utilize these material aspects—such as inviting the child to draw or using familiar objects like toys, videos, songs, and cartoon-themed bandages—to mitigate the child’s anxiety and discomfort. These practices not only provide comfort but also empower the child, fostering a sense of agency and participation in the vaccination process.

The study underscores that these material elements should be viewed as active “co-participants” in the vaccination experience. This perspective aligns with previous research that emphasizes the significant role of material objects in shaping caregiver-patient relationships (Lindén and Odenbring, 2022). Recognizing the importance of these often-overlooked ordinary objects is crucial, as they profoundly influence the vaccination process and interactions among the involved parties.

In light of these findings, we recommend that healthcare professionals receive training on the impact of materialities during vaccination encounters. Such training could enhance their understanding of how these elements affect vaccine decision-making and overall patient experience, thereby improving the effectiveness and comfort of the vaccination process.

## Notes

1. This can be attributed to the fact that all childhood vaccines are provided free of charge and are strongly recommended, though not mandatory (except for the tetanus and diphtheria vaccines). The national vaccination schedule begins at birth and holds particular significance during the first five years of life. However, it extends throughout an individual’s life, tailored to their risk profile and specific circumstances (Mendonça and Hilário, 2023).

2. “Vaccine hesitancy refers to delay in acceptance or refusal of vaccines despite availability of vaccination services. Vaccine hesitancy is complex and context specific varying across time, place and vaccines. It is influenced by factors such as complacency, convenience and confidence.” (MacDonald et al., 2015: 4163). This concept encompasses a broad spectrum of individuals, ranging from those who completely reject vaccines to those who harbor doubts about them.
